# Introducing biological realism into the study of developmental plasticity in behaviour

**DOI:** 10.1186/1742-9994-12-S1-S6

**Published:** 2015-08-24

**Authors:** Ton G G Groothuis, Barbara Taborsky

**Affiliations:** 1 Behavioural Biology, Groningen Institute for Evolutionary Life Sciences, Nijenborgh 7, 9747 AG, Groningen, the Netherlands; 2 Behavioural Ecology, Institute of Ecology and Evolution, University of Bern, Wohlenstrasse 50a, 3032 Hinterkappelen, Switzerland

**Keywords:** ontogeny, behavioural development, cumulative stress, match and mismatch, maternal effects, interaction effects, early environment, early programming, developmental plasticity

## Abstract

There is increasing attention for integrating mechanistic and functional approaches to the study of (behavioural) development. As environments are mostly unstable, it is now often assumed that genetic parental information is in many cases not sufficient for offspring to become optimally adapted to the environment and that early environmental cues, either indirectly via the parents or from direct experience, are necessary to prepare them for a specific environment later in life. To study whether these early developmental processes are adaptive and through which mechanism, not only the early environmental cues but also how they impinge on the later-life environmental context has therefore to be taken into account when measuring the animal's performance. We first discuss at the conceptual level six ways in which interactions between influences of different time windows during development may act (consolidation, cumulative information gathering and priming, compensation, buffering, matching and mismatching, context dependent trait expression). In addition we discuss how different environmental factors during the same time window may interact in shaping the phenotype during development. Next we discuss the pros and cons of several experimental designs for testing these interaction effects, highlighting the necessity for full, reciprocal designs and the importance of adjusting the nature and time of manipulation to the animal's adaptive capacity. We then review support for the interaction effects from both theoretical models and animal experiments in different taxa. This demonstrates indeed the existence of interactions at multiple levels, including different environmental factors, different time windows and between generations. As a consequence, development is a life-long, environment-dependent process and therefore manipulating only the early environment without taking interaction effects with other and later environmental influences into account may lead to wrong conclusions and may also explain inconsistent results in the literature.

## Introduction

Behavioural development has long been recognized as a key topic in the field of behavioural biology and is the subject of one of the four famous “why” questions of Niko Tinbergen [1]. It was also the topic of the famous debate about nurture or nature, which proved to be an irrelevant dichotomy [2] that is still surprisingly alive. In both frameworks behavioural development is mostly seen as part of the question about proximate mechanisms of behaviour. Indeed, how behaviour develops under the continuous interaction between genes, other internal factors and the environment, sparked by the recent interest in epigenetics, is an intriguing question. How sensitive phases and early organizing effects shape brain and behaviour, and to what extent there are constraints on developmental plasticity are highly relevant questions to understand normal behaviour, the causes of maladaptive behaviour and its potential treatments. However, in addition to these proximate approaches, the ultimate approach is equally relevant. Young animals are not just incomplete adults, but should have their own ontogenetic adaptations to their own special niche that might differ considerably from that of the adult. Although that niche is temporary, (mal) adaptation to it may have lifelong consequences. The relevance of the ultimate approach is now increasingly recognized. The increased attention for the study of development within a functional and evolutionary framework is at least partly caused by the interests of ecologists for development as a driving force for adaptation and evolution. They started to study how environmental cues experienced during development can give rise to different adaptive reaction norms. They also pursue the awareness for the adaptive importance of so called parental or maternal effects, in which the phenotype of the parent affects the phenotype of the offspring [3]. In essence, this is a pathway in which parents affect the environment in which the embryo or postnatal offspring develops, affecting its further development. Both maternal and direct environmental effects require the presence of developmental plasticity, in which the organism can develop into different phenotypes, depending on environmental cues, despite the presence of the same genes. Under some conditions, detailed below, parental effects provide the parents with a tool of transferring information to their offspring in a much more flexible way that their genes can do. Based on the experience the parents gather until or during reproduction they might be able to adjust offspring development to the prevalent or predicted environment in which the offspring will live. In many other situations, in which the offspring can gather the relevant information themselves, direct environmental influences take over the role of the “weather forecast” of the parents.

This functional framework has important consequences for the study of behavioural development. The standard approach to the study of behavioural development (and development in general) is to manipulate either genes or environment in an early stage of development, mostly under laboratory conditions, and then analyse its outcome on the behavioural phenotype later in life. In addition, often and deliberately only one specific gene or environmental factor is manipulated. Although this seems an obvious and clear-cut design, this approach may have several disadvantages either masking interesting results or leading to the wrong conclusions. Not only is the effect of the environment often depending on the genetic background and *vice versa*, so that the subtle interaction between both cannot be neglected [4]. But also in order to test the functional consequences of early influences for later life, one should take the later environment into account. It is the aim of this paper to (1) demonstrate that behavioural development is a lifelong process that includes an array of different interactions between environmental effects in early and late development and between different environmental effects, both indirect (parental effects) and direct and (2) to suggest alternative designs that, although not completely new, should be used more often.

## Development as a life-long interactive process of gathering information

The fact that studies on behavioural development often focus on the influence of early developmental phases is understandable as early in development the brain is not yet fully developed, probably most plastic and sensitive for changes in genetic or environmental cues. The idea of early sensitive phases in which environmental influences result in irreversible changes in behaviour was strengthened by the research in the previous century on filial and sexual imprinting, in which exposure to conspicuous objects in early life was demonstrated to have even effects on sexual partner choice in adulthood [5]. This notion was again strengthened by the flourishing research on song learning in songbirds in which during an early sensory phase auditory information is stored on the basis of which a bird somewhat later in the so called sensory-motor phase starts to practice its own song after which it becomes so called crystallized and fixed in its form [6]. This notion of early sensitive phases and irreversibility has perhaps been so appealing that later research, finding evidence for influences later in life, has had less influence. For example, for sexual imprinting it later turned out that the process consists also of a phase later in development, when the bird starts to reproduce. During its first courtship interactions in adulthood it again gathers information that either may consolidate or weaken the effects of experience in early life [7,8]. This second phase can be interpreted as a check of the earlier acquired information in the relevant context. In song birds there are many so called open-ended learners that acquire new song elements during their whole life, showing that sensitive phases are not restricted to early life [9]. In the study of development of sexual behaviour puberty has now become recognized as a sensitive phase for hormones and experience in addition to such a phase much earlier in life [10].

These examples demonstrate that development does not end before adulthood. This makes sense from a functional perspective. Experience early in life may not be an adequate predictor for circumstances later in life and therefore might need to be checked later in life in the adequate context. The environmental influences can be seen as sources of information for adjustment of development such that the animal becomes adequately adapted to its environment. This adaptation is not only relevant during adulthood, but also for earlier stages since early and juvenile mortality may be at least as strong a selection force as later reproduction. Therefore feedback from the environment might occur in many life history phases. When and which information is required will depend on the species, its life history and the predictability and relevant cues of its environment [11]. A recent analysis revealed that not only the extent but also the nature of predictability, either through seasonal variation or environmental autocorrelation, matters for phenotypic development [12]. In the extreme case of complete stable environments, development may need no environmental cues as development can be taken care of by genetic accommodation and no adjustment is needed. In case of complete instability and unpredictability, mothers may use the strategy of bet-hedging or offspring might become life-long plastic and sensitive for environmental cues.

The latter raises the question why organisms are not simply life-long plastic? This is a question about the potential costs of developmental and phenotypic plasticity. Costs might be related to the costs of rewiring the brain, of time it takes to adjust based on new input and disregard earlier information, of vulnerability to change based on incorrect cues, of missing the benefits of specialization and so on. Discussing the costs of phenotypic plasticity is somewhat outside the scope of this paper, and readers are referred to some excellent reviews on this topic [13,14], but see [15]. The potential costs of reshaping anatomy and morphology during development may be substantial, although obvious examples exist where this reshaping does occur, such as in some species of coral fishes where sex reversal in fish occurs regularly and seems to have clear fitness advantages [16]. However, adjustment of behaviour may need less costly reshaping, and therefore the study of the interaction between early and later environmental factors in shaping behavioural development becomes even more important for the study of behavioural development.

## Mechanisms of developmental plasticity in behaviour

### Maternal effects

The earliest source of information in development is via the mother. She can change the composition of the egg before fertilization, either by changing resources for the embryo such as nutrients, immune factors or vitamins, or signals such as hormones. Fathers can play a role here, too. Their genes may affect which genes of the mother come to expression via genomic imprinting. In addition, the quality of the father can affect the female deposition of resources and signals. This has been extensively studied in birds in which the influences of a variety of environmental cues on egg composition and size have been demonstrated (for reviews see [17,18]. Maternal effects on egg quality almost always end after oviposition in oviparous species although in fish species such as cichlids during parental care for the eggs urine containing parental hormones may enter the egg. This provides an opportunity for fathers too to affect egg composition. In placental animals there is a much longer and actually mutual exchange of substances between mother and offspring [19]. These prenatal parental effects are often overlooked and difficult to control for in studies manipulating postnatal environmental cures. Cross-fostering is sometimes claimed to control for maternal effects but disregards prenatal maternal effects.

One important topic associated with these maternal effects is the discussion whether mothers “actively or passively” bestow their eggs or embryos with resources and signals. In our opinion this is an inadequate dichotomy as “active” suggests a “decision” by the female (again a confusing word suggesting a conscious process) and passive suggests that the variation in maternal deposition is an inevitable consequence of environmental influences on her physiology. The real interesting issue here is to what extent mothers can regulate the deposition into eggs of resources and signals independent from what she would produce in her own circulation. This is discussed extensively in [20]. The current data suggest independent regulation as far as hormonal signals are concerned [21]. This would free the mother from a trade-off between allocation of these signals to her offspring and herself. This is likely to be different for resources such as antibodies and nutrients, unless these are available ad libitum. Clearly, for understanding functional consequences and trade-offs, we need to integrate proximate approaches with ultimate explanations.

Maternal and paternal effects can obviously occur in many ways also postnatally. An obvious pathway is via (pre- or) postnatal food provisioning that may provide the offspring a head start that has beneficial consequences throughout life, the so called ‘silver-spoon effect’ . In species in which offspring size or body mass is related to mother's size or body mass, this may translate in transgenerational effects (e.g. [22]). Such effects are also conceivable due to habitat imprinting, in which offspring choose their habitat to reproduce depending on what they have experienced when they were young as a consequence of parental nest site choice. Interestingly, such transgenerational effects are easy to misinterpret as genomic heritability but they are in fact non-genomic. These effects might be caused by epigenetic processes in which DNA expression is affected by environmental input, for example, by methylation or his tone modification that has to occur every generation again [23]. Many other maternal effects can be envisaged, ranging from food preferences, immune defences, and migration routes to partner preferences and tool use.

### Direct environment effects

The above discussed maternal or parental effects are often labelled as indirect environmental effects as the effect of the environment on the offspring is transmitted through the parents. Direct environmental effects have received more attention in the previous century (e.g. [24]) or have not been recognized as indirect environmental effects. In especially precocial species many environmental influences can be direct, although by determining the habitat in which the offspring experience these influences parental effects can be still playing a role. For example the level of early aggressive interactions between black-headed gull chicks affect their early endogenous testosterone production having long-term consequences for their sensitivity to the hormone later in life [25]. This early social experience is due to nest site choice of the parents so actually a parental effect. Also the social environment experienced early in life, such as social group size in cooperatively breeding species, is usually considered as direct environmental influence with potential strong effects on future social performance [26], but group size itself can be a function of parental quality. These examples suggest that environmental factors can constitute both direct and parental effects.

Similar to indirect (parental) effects, direct environmental effects can be induced by all possible features of the early environment including habitat (e.g., [27], food quality (e.g., [28], [29]) and availability or social context (e.g., [30]. These early, direct experiences often result in long-term preferences for similar features later in life as has been shown, for example, in experiments testing for habitat imprinting ([31]. Quality differences of the early environment such as early food conditions can exert long-term effects on fitness though carry-over effects [29,32], which work analogously to silver-spoon effects caused by parental provisioning (see above). Long-term effects induced by direct early experience can become manifest through stable reprogramming of gene expression profiles [33], which has been hypothesized to be caused by epigenetic modifications such as DNA methylation or chromatin changes.

### Interactions between early (in)direct environmental effects and later experience

Parental effects and effects directly induced by environmental conditions experienced early in life can, in our opinion, in many cases not be interpreted when the environmental effects on the offspring later in life are not taken into account. Below we sketch six scenarios in which the later experience can interact with early experience.

#### Consolidation

This process implies that the information gained during an earlier developmental stage only comes to expression in behaviour when in a later developmental stage the right experience is acquired. An example has been provided above in which the experience gained during exposure to adult conspecifics during the early sensitive phase of imprinting is strengthened by similar exposure later in life. This might be interpreted as a safeguard in which the relevance of the early information is checked in its appropriate context. If, however, the later context is not provided, the effect of the early information might not become expressed in behaviour [7,8].

#### Cumulative information gathering and priming

A mechanism that is related to the consolidation hypothesis is that early information is repeatedly checked and updated in the course of later life. This mechanism may be difficult to distinguish from consolidation. However, the consolidation hypothesis assumes that a limited amount of later experience ends the process whereas in the cumulative information gathering hypothesis the process is much more gradual and continuous. If one early exposure to a certain cue may provide an unreliable predictor for that aspect of the environment later in life, than it may be a good strategy to check and update this information more than one time. This mechanism and the conditions under which it is beneficial is discussed in the section “Support from theory”.

Cumulative information gathering is a more general formulation of the cumulative stress hypothesis [34] in which early stressors, via the activation of the HPA axis, makes the offspring increasingly vulnerable to subsequent stressors. This has also been called priming, in which a specific environmental cue makes the offspring increasingly sensitive for this cue later in life. An example outside the field of stress is that ducklings only learn to recognize the call of their mothers when the embryos have heard their own calls produced in the egg [35].

#### Compensation hypothesis

This hypothesis also comes from the stress literature in which exposure to early stress is mostly seen as aversive and not a preparation for later life. The idea here is that later experience may reduce the negative effects of the early stressors, such as later social support [36]. Another example might be compensatory growth when food becomes abundant after an initial period of low abundance. Such compensation might not fully compensate the initial detrimental effects or may come at a cost [37].

#### Buffering and the maternal capital hypothesis

In general, it is well conceivable that any information, acquired during early life, might have been inaccurate and that later conflicting information at the time the animal needs to express its behaviour might be more adequate and can weaken or overrule the effect of the earlier information. This assumes phenotypic plasticity in later life and will therefore depend on the costs associated with it. Others have suggested that the mother actually buffers offspring development to environmental influences during her reproduction (e.g. [38]. The idea here is that the environment is often unpredictable on the short run, and that previous experiences have cumulated over a much longer time period in the mother or even grandmother, so that not the direct environment during reproduction, but rather her long term physiological condition may be the best predictor for offspring programming. Nevertheless, a mismatch can occur when the later environment of the offspring differs from that in the previous generation(s). Buffering may also occur in direct environmental effects in which relying on only one environmental cue on one occasion might be risky. For further discussion see the section on “support from theory”.

#### Matching and mismatching

Early life influences may potentially act as predictors for the environment later so that, if the prediction is accurate, the offspring's phenotype “matches” the environment in which it will live, increasing its fitness. However, if the prediction is wrong, there would be a mismatch at the cost of the fitness of the offspring. Most of the work in this framework has been done on maternal effects. Maternal effects have been viewed in the past mostly as annoying noise for breeding programs. However, since the publication of the book by Mousseau and Fox [3] there is a strong tendency to view them as adaptive maternal programming: parents may provide a weather forecast for the environment in which the offspring will live and program their offspring for this environment. This has been formulated in the Predictive Adaptive Response hypothesis [39,40]. A consequence of this idea is that maternal effects, but also any other early direct environmental effect, can only be evaluated in an adaptive framework if the postnatal environment to which the offspring is supposed to be adapted to is taken into account. Moreover, using an environment for which the offspring was not programmed for may reveal only maladaptive effects. This may be the reason why effects of early stress are so often found to be detrimental. Effects of early stressors are often tested in later adulthood in standard almost stress-free rearing conditions, so that a mismatch exists between the early programming effects and the later environment. Moreover, the stressors used may be not at all those for which evolution has shaped the developmental trajectories. Therefore, testing this match-mismatch hypothesis can only be undertaken using environmental manipulations within the natural range for the species. Finally, selection on early programming may only work in case the environment is not stable but is predictable either within one generation (in the case of direct environmental effects) or from one generation to the other (in the case of parental or maternal effects). The extent of environmental predictability and instability over time is, however, often unknown, except in the case of cyclic phenomena such as seasonal variation [11]. For a discussion about the relationship between the match-mismatch hypothesis and other hypotheses see [41].

#### Context dependent trait expression

The difference in matching and mismatching results in the effect of early programming expressing itself differently depending on the context in which it is expressed later in life. This might be either because the same behaviour in one context is beneficial and in the other not, or because the behaviour is differently expressed in both contexts with either beneficial or detrimental effects. The match-mismatch hypothesis is therefore based on the functional consequences of either of these two options. However, it is conceivable that the second option, context dependent trait expression, may result in beneficial effects in both contexts. For example, elevated concentrations of testosterone in egg yolk stimulate the chick's begging behaviour for parental food but only when tested with chicks together with other chicks (when the relevant competitive context is present) and not when tested alone [42]. Other context dependent effects can occur if not early environmental information but genetic information generates context dependent effects. For example, human males having the less active version of the MAOA gene show, compared with those having the more active gene version, the most antisocial behaviour when having experienced maltreatment in their youth, but the lowest antisocial behaviour when not exposed to early maltreatment (see [43] for this and other examples).

## Approaches to study developmental plasticity

If we want to test how direct or indirect cues from the early environment are used by organisms to adapt to future conditions, we need to employ experiments investigating the importance of the early and late life environments. A number of experimental approaches have been used to pursue this aim, which follow a common general pattern. They provide organisms with two or more different environmental cues or conditions at an early developmental stage, either through the influence of parents or directly, and they test their performance at some later life-stage in conditions that are either correlated or uncorrelated with the early conditions.

**Figure 1:**
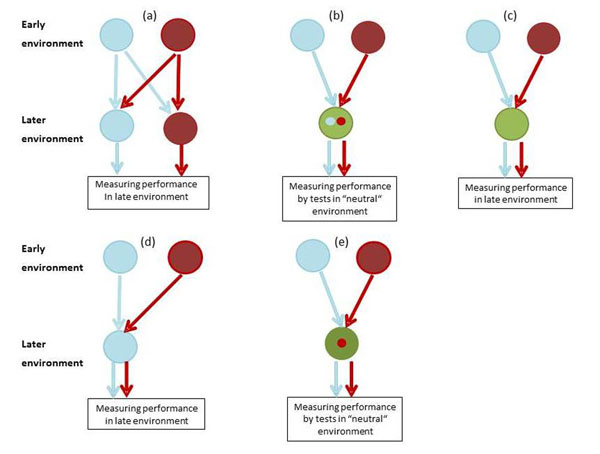
Schematic overview of five experimental designs to test developmental plasticity. The different colours of the large circles represent different environments. The small circles represent behavioural tests conducted within such an environment and which are aimed to represent tests related to the rearing environment. (a) Full cross-over design in which the control and experimental groups are split after initial rearing and further reared divided over both environments in a complete match-mismatch design. (b) Organisms are reared in either of two conditions, then transferred to a new condition (often standard housing and considered to be a “neutral” condition), in which the performance of both groups is measured by tests designed to reflect elements form the rearing environment. (c) After being reared in either of the two conditions, both groups are transferred to the same “neutral” holding condition, in which the performance of both groups are analysed without specific testing. (d) as in (c) but groups are transferred to only one condition (often the control condition). (e) As in (b), but here the performance later in life is analysed only by one common test in one condition (here, a condition related only to the experimental condition).

**Figure 2:**
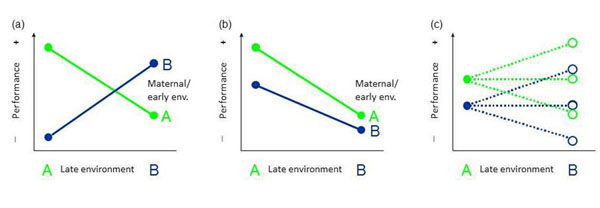
Early and late environments can influence the development of phenotypic traits (a) interactively (e.g., through environmental matching effects) or (b) additively (e.g., by carry-over effects). (c) Incomplete experiments, in which individuals are tested only in a single late environment, (here environment A) cannot distinguish between these two possibilities; dashed lines and open circles: possible outcomes if the performance in the later environment were also tested in environment B).

Most experiments on developmental plasticity follow one of the five designs depicted in Fig. 1. These approaches differ in the strength of inference that can be made with respect to the causal mechanisms of plasticity and its ultimate function. The strongest inference on the possible beneficial or detrimental effects of developmental plasticity can be made from full factorial experiments where early and late environments are manipulated reciprocally (Fig. 1a). Here both early and late environments are varied such that half of the experimental individuals are kept under identical conditions early and later in life, and the other half is switched between environments at the onset of the later-life treatment. By this reciprocal design it is possible to distinguish, for instance, interacting effects of early and late environment as expected by the match-mismatch hypothesis (Fig. 2a) from additive effects as they occur in the presence of carry-over effects (Fig. 2b). To get even closer to a relevant fitness estimate, reciprocal experiments should ideally be done under field conditions that is, in the presence of all important selective forces (see [44] for an excellent example in plants). For instance, [45] manipulated prenatal and postnatal food availability during both egg laying and the nestling stage. One of the interaction effects they found was that food supplemented chicks grew largest when their mothers were not supplemented during egg laying, suggesting that mothers had prepared their chicks via egg composition to use food resources more efficiently.

A second reciprocal design is typically used when studying the development of behavioural traits. By this design, often the hypothesis is tested that in different early environments animals develop different behavioural skills from which they can benefit when early and later-life conditions are similar. Here after being reared in different early environments animals are kept under identical, ‘neutral’ conditions until testing, that is, conditions that do not resemble any of the early environments. For testing, they are temporarily removed from these ‘neutral’ holding conditions and confronted with test situations that reflect the conditions in the early environments (Fig. 1b). Typical applications of this design are studies on the effects of the early social environment on later social performance. For example, zebra finches were kept in two social environments during adolescence (pair housed or group housed). During adulthood males from both treatments were temporarily brought into social settings resembling the pair housing (only females present, [46] or into a setting with mixed–sex groups [47]. Males performed better during social challenges that mimicked their respective adolescent social environments than during challenges of the opposite condition.

Many if not most developmental experiments are non-reciprocal designs, however. After being reared in two or more different early environments, organisms are kept and tested in one common environment which is often the standard laboratory housing condition and considered to be neutral (Fig. 1c) or resembles only one of the rearing conditions (Fig.1d). Often these experiments reveal highly interesting long-term or transgenerational effects such as impaired performance as result of a poor start in life [22,48,49]. However, functional consequences of this early programming effect can then not be tested as the performance of both groups may depend on the nature of the common later environment (Fig. 2c; dashed lines illustrate the possible outcomes if the performance in the later environment were not only tested in environment A, but also in environment B). For example, in the three above mentioned studies focal individuals from poor early conditions could have had a worse (carry-over effect), equal or even a better (benefits from matching) performance in a poor late environment compared to the other experimental group. Correspondingly, in studies of behavioural development, animal performance is often scored only in one late-life test, which is usually set up in the same behaviourally domain as the variation of the early environment (e.g., social challenges are used to test for influences of the early social environment), but not aiming at mimicking elements of one particular of the two rearing environments (Fig. 1e). These experiments are suited to test whether different skill levels result from different early environments *irrespective* of the type of late environment. For example, quite a number of such experiments were conducted in a social context. Most of these studies found that being reared in more complex early social environments results in improved social or reproductive skills later in life [50].

Clearly, in an experiment in which the performance of the animals is scored by means of staged behavioural tests (Fig. 1b,e), one cannot adequately test fitness consequences, which can only be done when the animals are kept in the adequate context for longer time periods [Fig. 1a,d, and 1c in case the later environment (green) is one that occurs naturally too].

Most developmental experiments require laboratory studies, for example, because a precise control of the environmental factor of interest is necessary that cannot be achieved under field conditions (e.g., control of temperature [51] or effects of individual diet are studied [28,52]). Developmental experiments in the laboratory, which are done with an evolutionary and behavioural ecology background, often propose functional explanations of the results and their fitness implications are discussed. For example, Henriksen and co-workers exposed female quail either to normal room temperature or high ambient temperature (mimicking hot summer) during egg laying and subsequently exposed the chicks of both mothers to both conditions. They found several interactive effects in physiology and behaviour of both the mother and chick environment, some of them suggestive for mothers being able to program their offspring for a hot environment [53]. In laboratory experiments it is usually impossible, however, to create natural conditions with all important selective forces in place. Most often natural predators and parasites are not present in the laboratory or, if predator effects are of core interest, they are presented behind barriers for ethical reasons [54]. This means that ‘fitness measures’ obtained in the laboratory have to be interpreted with extreme care for two reasons. (i) The phenotypic traits measured in later life must significantly affect fitness of our study species, a knowledge which can only be obtained by a thorough understanding of its biology in nature. It is not sufficient *a priori* to assume that certain traits such as body size, mass or growth rate will increase the fitness of any given study species. (ii) It is important to carefully choose the environmental conditions for the respective study species so that they reflect the natural conditions close enough to allow organisms to express evolved reaction norms, especially when one is not interested in pathological effects, but in testing to what extent developmental plasticity can help the organism to adapt to its environment.

Finally, it should be noted that the functional interpretation from the results of these designs can depend on what is seen as the experimental and the control condition. For example, studies that induced early stress in rodents by removing the mother from time to time from the pups may interpret this as the experimental condition and the non-removal as the control. However, in nature mothers can actually be frequently away to forage so that the experimental condition may actually be the control condition and vice versa.

## Environmental influences during multiple developmental windows

### Support from theory

Models on the evolution of adaptive plasticity generally agree on that plasticity requires at least three ingredients. A phase where organisms are sensitive to environmental cues that predict future conditions; a period where a plastic response is shown to match the forecasted conditions; and, finally, a phase when the produced phenotype is exposed to the selective environment that determines which phenotype has the highest fitness. Most models on adaptive plasticity allow these three ingredients to occur only once in a life history (reviewed in [55]). From empirical studies we know that this view is a gross oversimplification of the process of plastic development (e.g., [10,56]). Plastic adaptations to one state of the environment can be halted or even fully reversed when later in life individuals perceive cues signalling that environment has switched to the opposite state [26], and some organisms continue sampling and adjusting their phenotype to perceived environmental cues well beyond sexual maturity (e.g. [57] or even lifelong [58]). Thus these kinds of dynamics of plastic phenotypic development need to be taken into account in theoretical models if they should aid our understanding of developmental plasticity. The key conclusions of theoretical studies incorporating this complexity are summarized below.

In a first step towards modelling the dynamics of plastic development, reversible plasticity was considered [59]. Using predator-induced structures as example, a plastic response was modelled that allowed previously induced defence structures to be reduced again when predation risk vanished. The model specifically investigated the role of phenotypic response lags relative to the rate of environmental change. Such response lags are thought to help avoid overly quick responses towards incomplete or unreliable environmental information and thereby to save costs of plasticity. If response lags were short or the environmental information reliable reversible phenotypic plasticity readily evolved and the phenotypes closely tracked the state of the environment [59]. Time lags can be costly themselves, however, if they prevent an adequate response to a new environmental risk, e.g., the presence of a new predator. The model indeed predicts that if environmental information is incomplete and time lags are long the optimal strategy is to converge towards a generalist strategy that can cope with a broad range of environmental states, reducing plasticity.

The next step towards a more realistic view on development was taken by [55], who modelled development as a ‘constructive process’, where individuals adapt incrementally to their local environment. The authors explicitly model two strategies to be linked by a trade-off: (i) response lags, during which information about the environment is collected, or (ii) phenotypic specialization towards the current environmental state. At each of a defined number of time points, model organisms were allowed to choose between either promoting phenotypic specialization or sampling the environment without phenotypic change. The optimal strategy depends on the reliability of environmental cues. Spending more time specializing may in the end lead to a better adapted phenotype, but only if the initial estimate on the environmental state was correct. Spending more time sampling reduces the uncertainty about the state of the environment and may therefore avoid maladaptation. Longer sampling periods emerged when cues were moderately informative, but not when they were highly or weakly informative.

Finally, in a recent theoretical study optimal plasticity trajectories were analysed throughout the lifetime with organisms having full flexibility to repeatedly adjust their phenotype to one environment or reverse these specialisations to adapt to an alternative environment [60]. Organisms can sample and track the environmental states at different ages until death. All optimal reaction norms of phenotypic plasticity turned out to have a characteristic shape, which entails a broad region where after an environmental switch no or little phenotypic adjustments are made. This “plateau” of the reaction norm causes time lags of plastic responses as an emergent feature of the model. This suggests that response lags in phenotypic adjustment can be part of an optimal strategy rather than being caused by constraints. These response lags only vanish when plasticity costs are zero. Several common patterns were observed in a majority of model environments. (i) Typically there was an early-life peak of phenotypic adjustments once young individuals had accumulated sufficient environmental information. (ii) Then, after a period of reduced plasticity, a second, broader peak of plasticity follows in response to environmental changes during life time. (iii) Plastic adjustments at the end of life were rare, because the costs for such adjustments have to be paid, while the benefits are unlikely to be reaped before death. These results may at least partly explain the existence of multiple sensitive windows observed in empirical studies.

In summary, the reviewed models suggest that selection does not only favour an early single sensitive window with organizing effects, but also reversible plasticity under certain conditions, lagged responses to environmental change and multiple windows of enhanced plasticity during lifetime. The latter requires extended periods of time when organisms are sensitive to environmental cues. Extended sensitivity in combination with lagged responses open the possibility that organisms collect environmental information during multiple periods in life before they show a phenotypic response that integrates the different information they had collected. In the following sections we examine the ways how animals integrate information from different life stages when mounting plastic responses during development.

### Interactions between mother and offspring

In almost all research on prenatal maternal effects it is assumed that the embryo is just a slave of the mother in responding to maternal provisioning. In the human literature it is, however, well established that there is mutual exchange of substances such as hormones between mother and embryo affecting and manipulating each other in their own interest [19]. This substantially complicates the study of development. This is much easier in oviparous species in which the scope for interaction between embryo and mother is very limited after oviposition and can further be reduced by artificial incubation. There is recent evidence that in such species the embryo plays an important role, too. For example, embryos of turtle, fish and bird species are surprisingly capable of converting the maternal hormones to biological inactive or active components [61-63]. Moreover, prenatal influences such as incubation temperature affect the embryo's own hormone production. For example, incubation temperature affects hormonally guided sexual differentiation in the leopard gecko [64]. In wood ducks, incubation temperature affects the chick's own production of thyroid hormones, involved in growth and metabolism [65]. This begs the intriguing question to what extent the embryo might or might not “listen” to the maternal signals depending on its context [17]. Obviously, maternal effects may be so important because only the mother may have information about the best developmental pathway at this stage, but cues related to egg quality, incubation temperature, or vocalisations by other embryos might provide relevant cues for the embryo to determine what to do with the maternal signal.

Also after the embryonic phase direct experience of offspring can abolish or reverse maternal influences on behaviour. In the cooperatively breeding cichlid *Neolamprologus pulcher* dominant females produce smaller eggs in groups with many helpers compared to females in small groups [66]. Offspring born in small or large groups but reared separately from parents and helpers differed in their tendencies to show submissive and aggressive behaviour later in life. In contrast, siblings of these offspring reared for two months *within* their natal group also differed in later social behaviour, but showed exactly the opposite behavioural tendencies, suggesting that the direct social experience had fully reversed initial, maternal influences on behaviour [26].

### Interactions between early and late environment

As predicted by the model of [60], animals collect information from the environment repeatedly during multiple stages in life and use this additional information to correct or modify initially pursued developmental trajectories. Tracking of environmental change and reversal of early specialization might be expected to occur particularly in behavioural phenotypes as remodelling of behaviour is assumed to imply less plasticity costs than, for instance, morphological structures. Indeed there are examples where behavioural differences induced by early social experience are fully abolished if opposite experiences are made in the sub-adult stage. When laboratory rat pups experienced low levels of maternal care they expressed low-level maternal care as adults themselves. The effects of poor maternal social stimulation were fully abolished, however, by housing rats in socially enriched environments after weaning [67]. What looks like a reversal on the behavioural level may however also be a compensatory effect at the brain gene expression level [68]. The most prominent and efficient mechanism of information updating is learning. Guppy males (*Poecilia reticulata*) reared in visual contact with conspecific females developed a strong tendency to perform forced copulations, whereas those reared with visual contact to males developed overly long poor courtship displays. Both of these exaggerated behavioural tendencies were reduced to a ‘normal’ level after only 2 days of sexual experience as adults in direct contact with conspecifics [69].

Given the accumulating theoretical and empirical evidence that animals can collect and integrate environmental information across multiple life stages, experiments on development should now move a step forward towards more complex designs addressing explicitly the integrative abilities of developing organisms by including more than two developmental stages. A recent study is a promising example of such a more complex design [32]. In order to test the match-mismatch hypothesis, nutrition was manipulated at two levels at the larval and at the nymphal stage of burying beetles (*Nicrophorus vespilloides*) in a crossed design and the joint effects of these treatments on intra-sexual competitiveness were investigated in adult beetles again at two levels of competition. Larval and nymphal nutrition jointly affected male fighting success through indirect (larval) and direct (nymphal) silver-spoon effects on body size and fighting ability, respectively.

## Influences of multiple ecological factors

The majority of experiments that investigated environmental effects on plastic development in animals have varied a single environmental factor only, for example early resource quality [70] or quantity [29,52,71], competition [72,73], predation risk [74], social group size [26,75], or a single prenatal hormone or other egg component (see [76] for a review). Natural environments vary along multiple dimensions, however. In the wild, organisms grow up under the influence of a multitude of environmental triggers, which may act synergistically or antagonistically on phenotypic development. These environmental factors can influence development either simultaneously or successively during multiple sensitive developmental periods. To understand how natural environments shape phenotypic development is thus challenging, both with respect to experimental design and interpretation of results. If one environmental factor is varied at two levels there is essentially one reaction norm possible for a genotype, which has a positive, negative or zero slope. Already when we vary two environmental factors at two levels each, the number of possible combinations of reaction norms increases greatly and it becomes too large for testing as soon as three or more factors are varied (e.g., [77,78]. However, multiple environmental factors may only have additive or permissive effects or only specific independent effects on specific behaviours, reducing the number of combinations. Below we review how different maternal effects and different direct experiences can jointly shape developing phenotypes.

### Interactions between different maternal effects

Mothers provide their offspring with a wide array of resources and signals and the effect of one of these on the phenotype of the offspring might depend on others. For example, maternal stress in mammals and birds has an effect both on embryonic cortisol exposure as well as on blood and nutrient provisioning, making it hard to determine the relevant pathway explaining the effects on development [79,80]. In birds, mothers provide their eggs among many other things with immune factors, carotenoids and androgens that mothers may or may not adjust to each other [81]. It has been repeatedly demonstrated that testosterone in the yolk of bird eggs can be immunosuppressive for the embryo (for a review see [18]. It has been suggested that the effect of elevated androgen concentrations in the egg may be beneficial in good quality eggs but detrimental in poor quality eggs [17,82]. The same holds for eggs sired by good and poor fathers, [83]. Detrimental effects of yolk androgens may be counteracted by enhanced provisioning of maternal antibodies and carotenoids. Indeed, several studies have analysed correlations among different bird egg components, finding correlations that can differ both within populations, among females, and among populations. This suggests that mothers may be able to uncouple these components in an adaptive way (for a review see [76]. This obviously poses a challenge for experimental studies in which normally only one component is measured and manipulated and warrants many more experimental studies in which more than one component is manipulated.

### Interactions between different direct experiences

**Figure 3:**
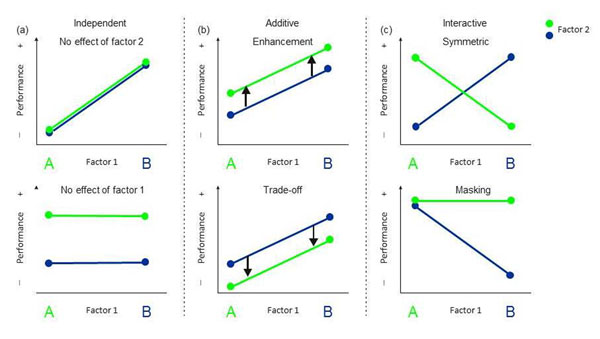
Typical examples of how two environmental factors can affect the development of phenotypic traits. The two factors affect trait expression (a) independently, (b) additively, where a second factor can enhance (upper panel) or reduce (lower panel) expression, or (c) interactively, where the two factors can contribute equally strong to phenotypic change (upper panel) or one level of one factor masks the effect of the other factor by a compensatory response; see text for examples of each case.

Generally, multiple environmental factors can have independent, additive or interactive effects on the development of phenotypic traits. Independent effects of the manipulated factors (Fig. 3a) occur quite often. Within the same experiment, different factors can independently affect different aspects of behavioural tendencies. For example, rats were reared in a balanced design either in isolation or in same-sex conspecifics groups and either in a barren or in an enriched cage. Isolation-reared rats had enhanced general activity as adults, irrespective of the physical enrichments during rearing, whereas, early enrichment improved habituation responses and spatial learning abilities of adults regardless of their social rearing environment [84]. The adaptive relevance of these results is unclear, however, as the rats may not have expressed evolved responses towards the highly artificial laboratory rearing conditions.

If we want to study evolved reaction norms in the laboratory, we should carefully think about the most important natural environmental factors for our study species, and vary them within the natural parameter range of these factors. For many animals two very important selective factors are food availability and predation risk, which can be connected through a trade-off limited by a common time constraint. Whether this potential trade-off is shaped by early experience has been studied in guppies (*Poecilia reticulata*) reared at two levels of early-life food abundance crossed with two levels of perceived predation threat [85]. Interestingly, anti-predator behaviour was *only* influenced by early perceived predation risk. Similarly, when juvenile cooperatively breeding cichlids (*N. pulcher*) were reared at two levels of social complexity crossed with two levels of perceived predation risk, predator avoidance behaviour later in life was only influenced by early predation threat [86]. The same early-life influences can trigger quite divergent long-term responses in males and females [87], however. Crickets (*Telogryllus commondus*) were kept as juveniles at two density levels crossed with three levels of male calling intensity. In females, the latency to respond to prospective mates was only influenced by the early calling environment. In contrast, male life span depended only on rearing density, whereas male age-specific calling effort was influenced by both factors interactively.

Growing evidence suggests that under certain conditions organisms may need joint information about multiple environmental factors in order to express adequate plastic responses during development [88]. For example, *Daphnia magna* required combined cues on current resource availability, photoperiod and population density in order to induce a switch from clonal reproduction to the production of sexual, dormant eggs, which can endure harsh conditions [77]. Additive effects of two factors become apparent by parallel reaction norms, but they can be interpreted in two ways; a second factor can be viewed to enhance the effect induced by a first factor (Fig. 3b, upper panel) or to reduce its effect (Fig. 3b, lower panel). The latter case may indicate that a trade-off is involved between the responses to both factors. An example of an enhancing effect is that the presence of early predator cues enhanced growth and thus final size in both low-food and high-food reared guppies [85]. An example of a trade-off is that anti-predator responses may have to be traded off against the development of morphological structures. In the absence of predation risk, wood frog tadpoles (*Rana sylvatica*) develop increasingly longer guts with increasing conspecific competition [89]. The same positive relationship between gut length and competitor density was found if tadpoles were reared in different concentrations of predator cues, but the higher the perceived predation risk the shorter the gut length was across all density treatments. Instead, under predation risk tadpoles allocated resources more into tail development.

Certainly the most interesting outcomes of designs with multiple environmental factors are interactive effects where reaction norms differ in slope. Most striking are examples where the slopes of reaction norms have opposite signs (Fig. 3c, upper panel). This was found, for example, when the influences of early social and predator experiences on the social behaviour of the cooperatively breeding cichlid *N. pulcher *were investigated. Different behavioural specializations in the social domain of the four treatment groups indicated that these fish required the information of both early environmental experiences to develop adequate behaviours [86], a fact that was overlooked when only the early social environment had been manipulated [28,76,90].

Unless full factorial experiments are employed, environmental effects may be entirely overlooked because they can be masked by a second factor. This can happen when one reaction norm has a slope different from zero and the other one is flat (Fig. 3c, lower panel). This has been demonstrated in crickets (*Telogyllus commodus*), in which a protein reduced diet resulted in the development of smaller adult size. These size differences vanished, however, when crickets were provided with acoustic cues signalling high future intra-sexual competition. By anticipating future competition, crickets enhanced growth during their last nymphal stage and were thereby able to compensate fully for initial grow deficits [91]. Interestingly, also the absence of an environmental trigger can have masking effects. Tail morphology of tadpoles is a textbook example of predator induced plasticity. When the population density of tree frog tadpoles (*Hyla femoralis*) was varied, no plastic response in tail morphology was observed, although low-densities are an indicator of higher potential predation risk. As soon as chemical predation cues were added, however, tail morphology readily varied with population density as predicted [92].

## Multiple ecological factors acting during multiple developmental stages

From the evidence reviewed above it is only a small step to predict an even more complex process namely that multiple environmental triggers will jointly influence development at multiple ontogenetic stages or across generations. Experiments investigating effects of multiple triggers during multiple stages will, in the simplest case with all factors being varied at two levels, yield 16 possible phenotypic outcomes. Such a design has been done in a well-recognized study on environmental induction of resting egg production in *Daphnia pulicaria *[93]. In this species a reliable information on the seasonal stage, which can only be estimated from resource availability *and* photoperiod, is of utmost importance to avoid the high costs of too early (loss of productivity) or too late (risk of extinction) resting egg production. Therefore the authors assumed that long-term information obtained by the maternal generation may improve the offspring's estimate of seasonal stage, and that offspring will use a combination of maternal and own conditions to decide about resting egg production, which was exactly what was found in this experiment.

## Conclusions

We reviewed evidence showing that simple developmental experiments manipulating the environmental conditions at only one ontogenetic stage and/or considering only a single component of the environment will not allow us to understand whether and how early parental or direct environmental influences can generate adaptive phenotypic plasticity. Moreover, results of such incomplete designs can be at best shed light on some causal processes, but can also lead, due to context dependent effects and masking, to misleading discrepancies in the literature. Here we argue that multiple and intricate interactive external and internal influences at the levels of different environmental factors, different time windows and between generations often determine the developmental trajectories of organisms, leading to well-integrated adult phenotypes. Therefore the field of developmental plasticity should move towards more realistic, complex experimental studies which better reflect the natural conditions organisms have been adapted to during the course of evolution. A thorough knowledge of a study species' life history, together with available results from correlative field studies, leading to better predictions about its important sensitive windows, key selective ecological factors and the environmental variability and predictability the species has adapted to should be obtained before setting up more informed laboratory experiments in the future. Obviously more complex developmental experiments may require larger sample sizes to maintain statistical power, are likely to be more time consuming, and they will reveal more complex reaction norms with less straightforward interpretations as compared to single-factor experiments. The gain in terms of an improved understanding of the intricate interactions between environment and phenotypic development by such designs is considerable, however, which readily outweighs these costs.

## Authors’ contributions

Both authors contributed equally to this paper.

## Declarations

Publication costs for this article were funded by the German Research Foundation (FOR 1232) and the Open Access Publication Fund of Bielefeld and Muenster University.
